# Real-time open-source FLIM analysis

**DOI:** 10.3389/fbinf.2023.1286983

**Published:** 2023-11-30

**Authors:** Kevin K. D. Tan, Mark A. Tsuchida, Jenu V. Chacko, Niklas A. Gahm, Kevin W. Eliceiri

**Affiliations:** ^1^ Department of Biomedical Engineering, University of Wisconsin, Madison, WI, United States; ^2^ Center for Quantitative Cell Imaging, University of Wisconsin, Madison, WI, United States; ^3^ Morgridge Institute for Research, Madison, WI, United States; ^4^ Department of Medical Physics, University of Wisconsin, Madison, WI, United States

**Keywords:** fluorescence lifetime, FLIM, real-time FLIM analysis, open source, phasor, napari, live FLIM

## Abstract

Fluorescence lifetime imaging microscopy (FLIM) provides valuable quantitative insights into fluorophores’ chemical microenvironment. Due to long computation times and the lack of accessible, open-source real-time analysis toolkits, traditional analysis of FLIM data, particularly with the widely used time-correlated single-photon counting (TCSPC) approach, typically occurs after acquisition. As a result, uncertainties about the quality of FLIM data persist even after collection, frequently necessitating the extension of imaging sessions. Unfortunately, prolonged sessions not only risk missing important biological events but also cause photobleaching and photodamage. We present the first open-source program designed for real-time FLIM analysis during specimen scanning to address these challenges. Our approach combines acquisition with real-time computational and visualization capabilities, allowing us to assess FLIM data quality on the fly. Our open-source real-time FLIM viewer, integrated as a Napari plugin, displays phasor analysis and rapid lifetime determination (RLD) results computed from real-time data transmitted by acquisition software such as the open-source Micro-Manager-based OpenScan package. Our method facilitates early identification of FLIM signatures and data quality assessment by providing preliminary analysis during acquisition. This not only speeds up the imaging process, but it is especially useful when imaging sensitive live biological samples.

## 1 Introduction

Fluorescence lifetime imaging microscopy (FLIM) maps the spatial distribution of excited state lifetimes over a field of view, generating contrast that is in addition to the fluorophore (intensity) distribution. Fluorescence lifetime measures the inverse of the rate of decay of fluorescence after excitation (van Munster et al., 2005; Datta et al., 2020). The measurement of fluorescence lifetime can provide a unique sensitivity to measurements of variations in the microenvironment of fluorophores and the physical structure of the molecule (Bastiaens and Squire, 1999; Chen et al., 2013; Datta et al., 2020). For example, FLIM is used to monitor the fluorescence lifetime of free and enzyme-bound reduced nicotinamide adenine dinucleotide in live cells and correlate the ratio with the metabolic state of the cells (Bird et al., 2005; Chacko and Eliceiri, 2019; Datta et al., 2020). FLIM measurements can be performed in the time domain or frequency domain. A principal method of time-domain FLIM employs time-correlated single-photon counting (TCSPC) to measure per-pixel photon histograms binned by time delay relative to pulsed excitation.

From such photon histograms, fitting to an exponential or multi-exponential decay function is a common method for determining fluorescence lifetime. More specifically, non-linear least-squares fitting via the Levenberg–Marquardt algorithm (LMA) is the standard in TCSPC-FLIM (Becker, 2021). However, LMA computation is usually slower than acquisition speed, so a real-time per-pixel computation of fluorescence lifetime during imaging (∼100 kHz pixel rate) is not feasible. Therefore, unlike fluorescence intensity, which is displayed and analyzed in real-time, fluorescence lifetime is typically computed and analyzed after acquisition in an offline analysis procedure (Becker, 2005). As a result, it is very common for users to find out only at a later time whether their data collection accurately recorded meaningful changes in fluorescence lifetimes. The lack of real-time visualization of FLIM undermines live-cell imaging applications when the microscopist would benefit from visual feedback for determining the length of the experiment or controlling other experimental parameters. Real-time FLIM (RT-FLIM) results would enable users to correct issues, observe dynamic systems, or locate regions of interest (ROIs) for more detailed imaging. Furthermore, real-time analysis opens the door to more complex experiment design that might require real-time feedback.

Currently, there are a few closed-source options for RT-FLIM, most that are part of commercial systems. One recently added and popular option for RT-FLIM for laser-scanning microscopy is the commercially available closed-source Leica FALCON system (Alvarez et al., 2019). Other FLIM hardware vendors such as Becker-Hickl, PicoQuant, and ISS have their own applications that support a version of real-time FLIM (Liao et al., 2014; Orthaus-Mueller et al., 2016; Becker, 2021) and custom laboratory-made applications (Laptenok et al., 2007; Klemm et al., 2015; Zhang et al., 2021). Unfortunately, there are currently no open-source options for RT-FLIM, severely limiting access and also preventing code iteration by other FLIM laboratories.

Here, we present an open-source RT-FLIM viewer that leverages existing open-source components such as the FLIM analysis library FLIMLib (Gao et al., 2020) and the visualization package Napari (Sofroniew et al., 2022). Our viewer, named Napari-Live-FLIM, is a Napari plugin which, in principle, can be made to interface with any producer of TCSPC-FLIM histogram images. We demonstrate its use with the open-source laser scanning acquisition software OpenScan (https://eliceirilab.org/openscan), which, in turn, operates as a module for the general microscope image acquisition software program Micro-Manager (Edelstein et al., 2010; Edelstein et al., 2014).

## 2 Materials and methods

We developed our live, interactive FLIM viewer (Napari-Live-FLIM) in Python as a plugin for the Napari multi-dimensional image viewer (Sofroniew et al., 2022). The viewer provides a user interface that is updated as new results are available from incoming image frames. We employ standard Python multithreading techniques to perform the FLIM analysis in the background; results are cached where possible to increase responsiveness.

To transfer FLIM histogram data from acquisition software to Napari-Live-FLIM, we use a temporary file for each frame, together with simple user datagram protocol (UDP) messages to notify the viewer of the availability of a new frame. By memory mapping the temporary file on both the sender and receiver sides, large amounts of data can be transferred efficiently.

We use FLIMLib (FLIMLib, 2022) for lifetime analysis using LMA, RLD, and phasors. Because FLIMLib is a C library (with Java bindings), we first developed Python bindings for FLIMLib so that the necessary functions could be called. This included adding multi-pixel fitting functions to FLIMLib to facilitate fast analysis by avoiding looping over image pixels in the Python code. Python bindings were created using the ctypes standard library module with a hand-written wrapper code to check inputs and facilitate usage with NumPy arrays as input and output. These bindings are general-purpose and can be used for FLIM analysis outside of Napari-Live-FLIM; we released them as a separate Python package (flimlib) with documentation and unit tests.

The laser scanning microscope (LSM) and fast-timing electronics used for these tests have been previously described (Chacko and Eliceiri, 2019). The software program used for controlling the data acquisition card and laser scanning hardware called OpenScan (unpublished) is available to download from https://github.com/openscan-lsm/OpenScan.

## 3 Results

### 3.1 Choice of analysis methods for real-time FLIM

RT-FLIM is primarily focused on time domain-based FLIM methods. Most of these time domain methods involve single-photon counting (SPC); thus, the critical parameters to optimize for in a FLIM viewfinder are fast processing and informative rough lifetime estimation from very low photon counts (Becker, 2021). As such, to find an appropriate real-time methodology which can act as a lifetime viewfinder, we compared four common fast-FLIM techniques, namely, rapid lifetime determination (RLD), phasor analysis, Laguerre deconvolution, and noise-corrected principal component analysis (NC-PCA). We benchmarked the relative speed and performance cost of these four algorithms, and we found that RLD was the fastest among the tested methods that provide single exponential lifetime estimates. We also determined that phasor analysis is similarly fast and uniquely able to provide multicomponent analysis for multiexponential models in a graphical plot known as a phasor plot (Ranjit et al., 2019). The results are highlighted in the supplementary methods, however not discussed in detail in this article. We decided to implement both RLD and phasor approaches in RT-FLIM as analysis options due to their simplicity and good performance. Phasor plots and 2D-phasor analysis are powerful tools for visually separating species with separable fluorescence lifetime distributions in an image (Datta et al., 2020). We chose phasor analysis aided with RLD as a comprehensive solution for RT-FLIM analysis.

### 3.2 Napari-Live-FLIM architecture

**FIGURE 1:**
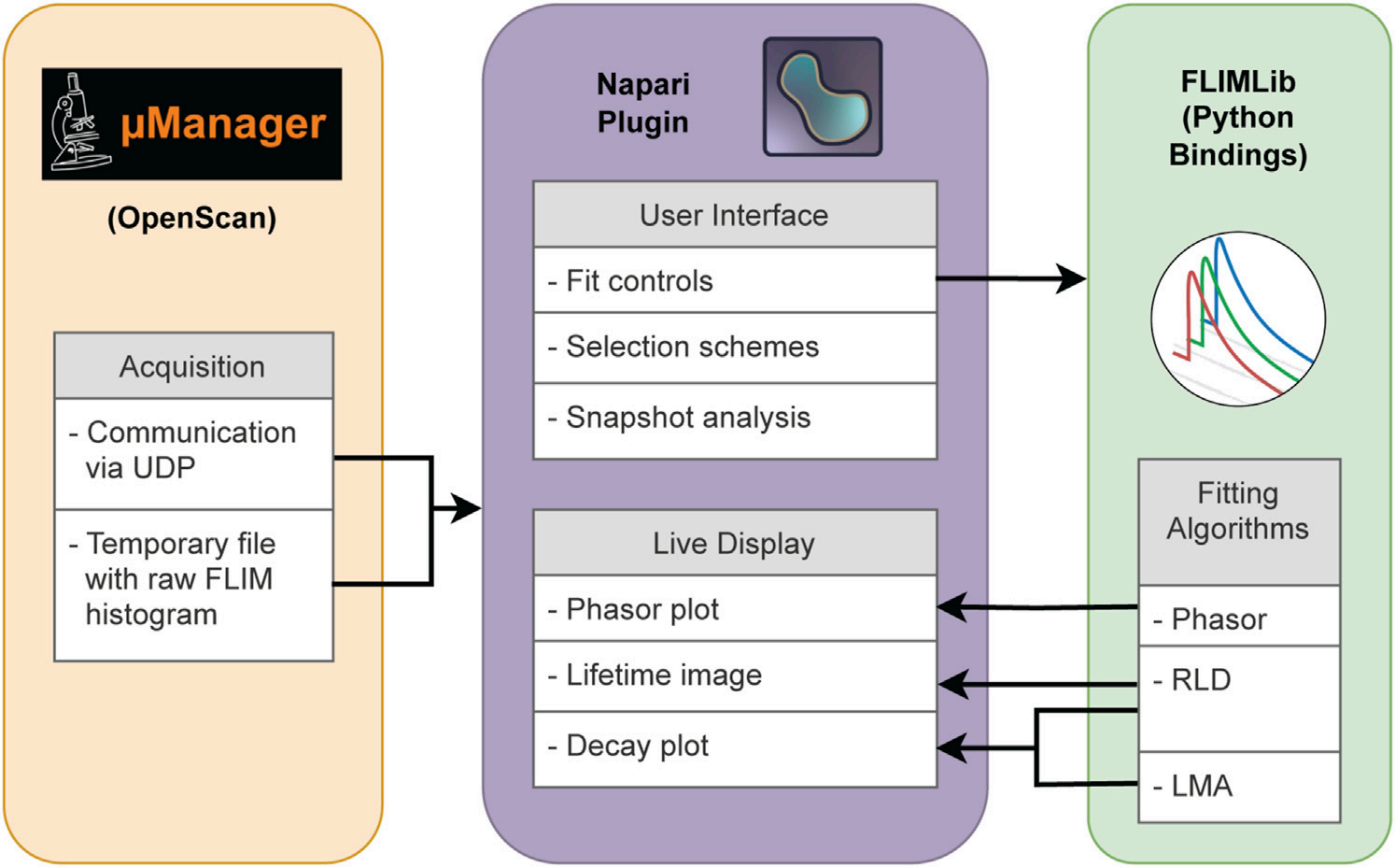
Software schematic representation of Napari-Live-FLIM. Raw FLIM histogram data are sent from OpenScan to Napari-Live-FLIM via temporary files and signaled by UDP messages. Napari-Live-FLIM provides user interaction and visualization of FLIM results which are calculated in real time via the FLIMLib Python bindings.

Figure 1 shows the overall architecture of Napari-Live-FLIM, our real-time FLIM viewer. We chose to develop Napari-Live-FLIM to run as a separate program from the acquisition software applications (Micro-Manager and OpenScan) so that it can potentially be attached to different acquisition software programs. In addition to being a workaround for the lack of support for FLIM datasets in Micro-Manager, this scheme also permitted us to take advantage of NumPy and Napari, which facilitated rapid development of the analysis, display, and user interface.

In order to conduct FLIM analysis in real time, the computation steps must be short and fast while maintaining a high-level user interface with a minimal learning curve. We chose to handle all computation of FLIM results within the high-performance C programming language and extend the user experience in Python. The other motivation for choosing the C program was to utilize FLIMLib (FLIMLib, 2022), an existing open-source C library for FLIM data analysis co-developed by our group. FLIMLib has an implementation of LMA and many other popular FLIM analysis methods.

### 3.3 Napari-Live-FLIM user interface

**FIGURE 2:**
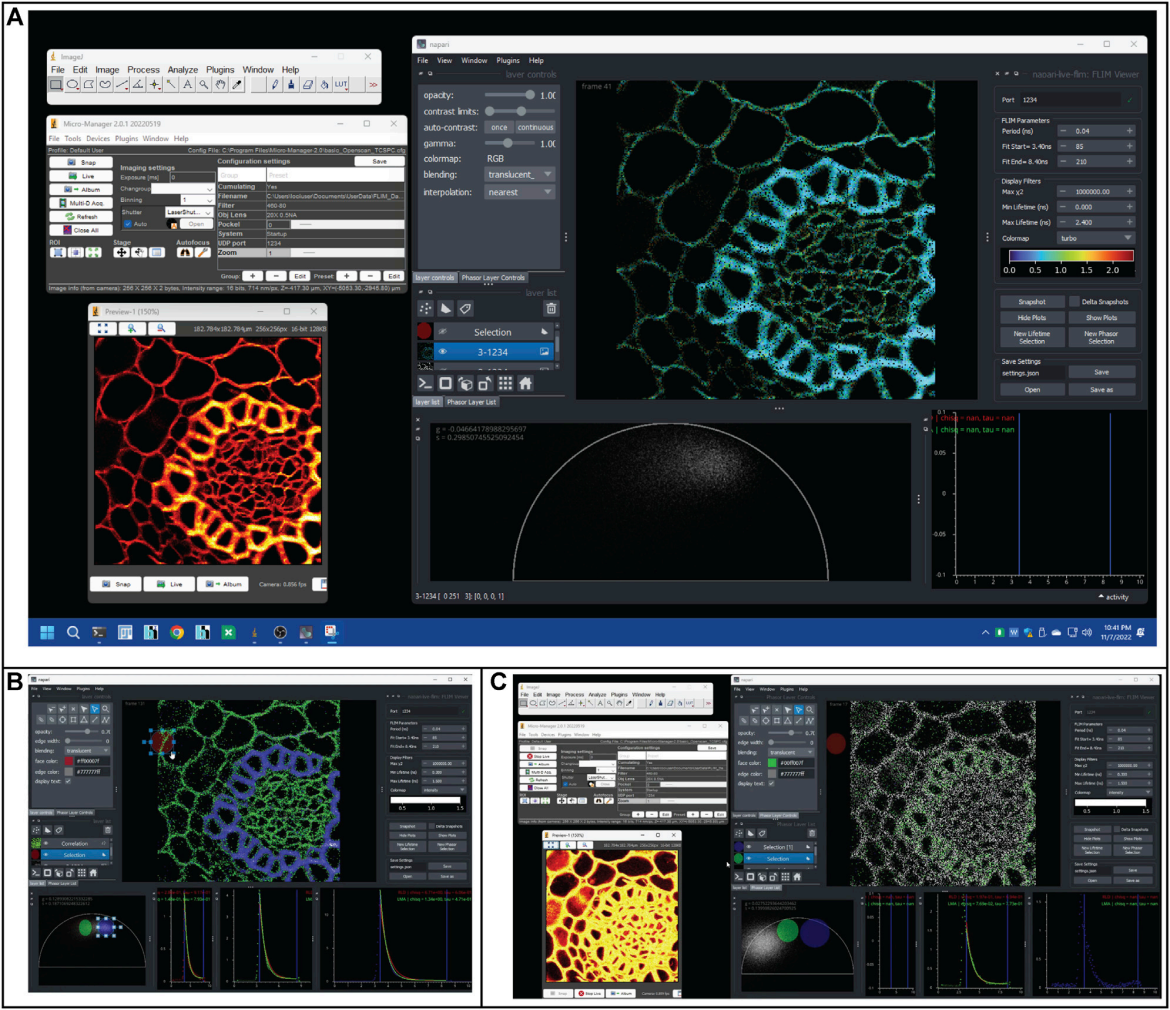
Selections of screenshots show how to use phasor-based selection to identify FLIM artifacts during a real-time acquisition. **(A)** Screenshot of OpenScan/Micro-Manager and Napari-FLIM-viewer operating in real time. The Micro-Manager window shows the essential OpenScan controls and a Preview window that shows the intensity image (red hot color). The Napari window shows the fitted lifetime image (turbo colormap) and the phasor distribution. The colors show two ROIs with different lifetime values (green and cyan). **(B)** The two ROIs are separated using the phasor cursor selection as blue and green cursors (cursors are shown in the phasor tab and corresponding colored pixels in the image). **(C)** A pile-up artifact generates a clear intensity image (seen in the Micro-Manager Preview window), but the FLIM data are skewed, seen by the mislocated phasor distribution and lack of expected pixel colors in the selected phasor cursors.

**FIGURE 3:**
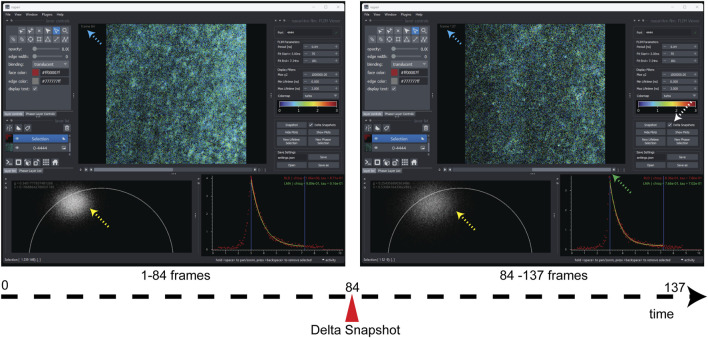
Snapshots of screenshot comparison here show the two snapshots from a single FLIM live acquisition, which are viewed by dragging the scrollbar (green dashed arrow). The delta-snapshot (white dashed arrow) features temporal slicing of incoming FLIM data into different FLIM sub-datasets that can be compared real time to gain an understanding of signal strength, phasor plot density (yellow dashed arrows), and improvement in fitting accuracy and any changes in the sample that correspond to that section of FLIM data stream. The blue dashed arrows show the current frame number, and the green arrow shows the scrollbar which facilitates visualization of the two temporal slices. Note that the screenshots were made post-collection.

Napari-Live-FLIM is a Napari plugin downloadable from the Napari Hub. The plugin offers a variety of interactive controls and displays for real-time FLIM analysis (Figures 2, 3). The Napari-Live-FLIM plugin’s main display shows the RLD result in the form of a lifetime image. The lifetime image is colored by the estimated lifetime at each pixel and scaled in brightness by the pixel’s intensity. Below the lifetime image is a phasor plot for visualization of phasor analysis results. To the right of the phasor plot are decay plots showing raw data and curve fitting results within selected regions. To the right of the lifetime image is a widget containing the user controls for the Napari-Live-FLIM plugin. The controls for FLIM analysis include the range of bins in the FLIM histogram to use in analysis and the time resolution. Additionally, users are able to threshold pixels in the lifetime image based on fluorescence lifetime and the chi-square (𝜒^2^, metric for goodness of the fit/model) statistic of the RLD estimate. Another user-defined parameter is the colormap used for the lifetime image. These parameters can be saved to a JSON file to be loaded later for a post-acquisition analysis.

An important feature we added to allow users to interact with and dissect the FLIM data is the ability to create selections of certain ROIs within the lifetime image or clusters of phasors within the phasor plot. Selections are made possible through the use of a Napari shapes layer. Shapes layers allow users to create various selection areas such as ellipses, rectangles, and polygons, and manipulate their size and position on the display. The screenshot presented in Figure 2A shows a view of the plugin. Separate, color coordinated selections can be created, reorganized, hidden, and deleted using UI (Figure 2B). Pixels selected in the lifetime image are highlighted within the phasor plot, and conversely, phasors selected in the phasor plot are highlighted in the lifetime image (Figure 2B). This correlation feature allows users to select ROIs and determine where they lie on the phasor plot and, in reverse, visualize where certain phasors occur on their sample. By linking these two views, it may be possible to make unique, more confident observations of the data. As a demonstration (Figure 2C), we can visualize “pile-up” artifact in real-time FLIM data (phasor distribution) even when intensity data fails to see any difference. Lastly, with these selected regions, additional analysis can be performed. By averaging the FLIM histogram data within the selections, analysis can be reduced to a single fit. Therefore, it is possible to run the slow gold standard LMA fitting algorithm on this averaged data. This LMA implementation may be useful to confirm observations made by RLD and phasor against what can be considered ground truth. Using these averaged histogram data, the LMA and RLD fitted decay curves are displayed on 2D intensity–time axes. Refer to the demonstration of real-time FLIM in Supplementary Video S1. The data itself are also plotted as a scatter plot, and two vertical lines delineate the user-defined fit range. In the upper right of these decay plots, the numerical results of the LMA and RLD fits are displayed. A major use for the decay plot is to observe the trends in the FLIM histograms in order to assess the quality of the data and set reasonable fitting parameters.

Another useful feature that we added to the Napari-Live-FLIM application is the ability to store temporal snapshots of the FLIM data as it arrived. Being able to compare time points in the acquisition is necessary in order to monitor the quality of data as they accumulate and to analyze a specimen that is changing in real time. Clicking the “Snapshot” button creates a new snapshot of the current histogrammed FLIM data (Figure 3). These snapshots can be revisited by sliding the scrollbar underneath the lifetime image (green arrow in Figure 3). Snapshots are ordered from oldest to newest; the last snapshot being the one that is reserved for live data. All snapshots can be analyzed in the same manner, allowing for easy comparison. Supplementary Video S1 shows general working using Napari-Live-FLIM and a demonstration of the use of the “Delta snapshots” option during a real-time acquisition (at time 3:29). Figure 3 compares two snapshots created on live neuronal culture, separating FLIM by a temporal breakpoint (associated with a different z-selection in the culture) without stopping or pausing multiphoton laser scanning for slicing the collection. This is critical for monitoring the FLIM response to drugs or sample treatments on the fly.

### 3.4 Usage and performance

All the described software applications are open-source and available at https://github.com/uw-loci/napari-live-flim. For ease of review and testing, we have provided a sample dataset and simulation of the replay option to readers to test our plugin. The sample data are available at Example Dataset (for replay test), and a live demonstration on how to use the sample data is shown in Supplementary Video S2.

A detailed guide on how to set up Napari-Live-FLIM with OpenScan is provided in the Supplementary Materials. We provide a video demonstration on how to set up OpenScan and Napari-Live-FLIM for real-time visualization (Supplementary Video S3). We also demonstrate live-chlorophyll fluorescence from *Arabidopsis thaliana* samples (Supplementary Video S1).

Performance: We tested the overall speed of Napari-Live-FLIM to make sure that it is capable of keeping up with imaging. We ran benchmarks by sending a test FLIM dataset with 50 frames, each with 256 × 256 pixels and 256 time bins at a rate of two frames per second (Table 1). These benchmarks were performed on a 64-bit Windows PC with an Intel Core i5-7300HQ 2.5 GHz CPU, 8 GB of 2,133 MHz RAM, and 2 TB NVMe PCIe 4.0 Gen4 x4 Solid State Drive. A total of 49 frames were processed by Napari-Live-FLIM, which computed a lifetime image and phasor plot on separate threads. The median time it took between the arrival of data and display of results was 124 ms. The median time that was taken to create the lifetime image and phasor plot was 116 and 34.5 ms, respectively. Since a typical scan rate for TCSPC FLIM with this image size is about 300 ms per frame, we conclude that our analysis can keep up with most acquisitions (Becker, 2021). Due to the way we queue incoming data, we ensure that we remain synced with acquisition by only processing the most recent accumulated frame. This occasionally results in a frame not being processed, which explains why only 49 out of the 50 frames were processed in our test. It should be noted that utilization of our selection feature creates some overhead, especially with large selection areas and many selections. However, since these tasks run on separate threads, we found that for reasonable selections, the added computational load is manageable.

**TABLE 1:** Benchmark table for live-FLIM imaging.

Computation step after arrival of new data	Median time (ms)
Lifetime image (RLD)	116
Phasor plot	34.5
Overall processing time of new frame	124

## 4 Discussion

Phasor-based contrast in FLIM has been attributed to frequency domain techniques for the past couple of decades, and it mostly belongs to a single commercial vendor (Liao et al., 2014; Ranjit et al., 2018). Many instances of academic solutions were proposed in the period that could generate phasor plots but were limited in scope due to closed source codes that severely limited sharing and extensibility (Yaseen et al., 2017; Schrimpf et al., 2018; Sorrells et al., 2021). Napari-Live-FLIM is the first open-source solution to handle live phasor plots and provide n-dimensional filtering on a real-time FLIM acquisition. This easily installed and customizable plugin allows microscopes to be set up for real-time FLIM analysis. Predetermined values for FLIM collection from the hardware can make the operations easier and make Napari-Live-FLIM suitable for microscopy cores that allow FLIM imaging facilities to users. RLD-based analysis, however, has a lot of open-sourced solutions and provides straightforward computation but is limited to a smaller group of users who use the FLIM technique in on-board implementations of FLIM analysis for multipixel field-programmable gate arrays (FPGAs) and CMOS/FLIM cameras (Henderson et al., 2014; Zang et al., 2023). Napari-Live-FLIM proposes a unique RLD-based live estimation of pixel-wise lifetimes that offers a preview of the FLIM image, aided with simultaneous phasor plots that can address the distribution of lifetimes and quality of the FLIM data.

Napari-Live-FLIM offers ROI-based fitting that enables users to fit a group of pixels of interest, brought together either by phasor or spatial features. The fitting is performed in LMA and generates a confidence map that could allow the user to determine the collection time for their experiment. Integrating the method with Napari makes it possible to integrate other image analysis routines into FLIM analysis and deploy them in a real-time manner for feedback. For example, the use of 3D filters and segmentation can separate regions of interest and allow users to focus collection on their interests. This is important as many GPU-based image optimization algorithms are readily available in Napari (https://github.com/haesleinhuepf/napari-cupy-image-processing) and through community-driven napari-hub. Moreover, an image analysis pipeline can easily be integrated into the napari-console for exporting FLIM outputs for analysis outside Python.

The different parts of our open-source software applications, FLIMLib, OpenScan, and Napari-Live-FLIM are modular and could be improved, upgraded, and updated without affecting each other. Current tests we demonstrate are using the OpenScan framework that supports laser scanning microscopy under the Micro-Manager program. However, this bridge is completely modular, allowing users to adapt the viewer to other FLIM control systems.

The key feature of our library is the open-sourced architecture, which is built upon all open-sourced components. Program solutions such as FlimLib and FLIMJ offered an open-source FLIM analysis solution that could carry out fitting and exponential library analyses but was limited by the inherent limitations of the programming language they were written in. Extending FlimLib to Python for exponential curve analysis encourages the larger Python-based scientific community to read and contribute to the project and the progress of FLIM-based analysis tools. However, the exponential fitting methods used in the project are not limited to FLIM and can be extended to non-FLIM applications that could use fast real-time analysis of exponential curves or arrays of exponential curves.

## 5 Conclusion

We present an open-source tool for real-time analysis of FLIM data during acquisition called Napari-Live-FLIM. Currently, it is integrated with OpenScan and available as a Napari plugin. This tool enables efficient and informative data collection of FLIM. Moving forward, this tool can be improved through further code optimization such as increased threading for parallelization and the support of specific hardware to handle different data acquisition setups. Upgrades of the application will enable higher-throughput FLIM systems. Currently, Napari-Live-FLIM provides phasor and RLD analysis of the data, but due to its open-source nature and the potential of the methodology, it is set up to be readily expandable with alternate analysis methods.

## Data Availability

The codes and sample-data presented in the study are publicly available on Github and Zenodo. Additional testing details can be found in the Supplementary Material. Code: https://github.com/uw-loci/napari-live-flim. Data and demonstration videos: https://zenodo.org/records/10019525.
