# Supplementary material for: Real-time open-source FLIM analysis
Source: Front Bioinform. 2023 Nov 30;3:1286983. doi: 10.3389/fbinf.2023.1286983 (PMC10720713; doi:10.3389/fbinf.2023.1286983)
Supplement: Supplementary file 1 [file DataSheet1.docx]

**Real-Time Open-Source FLIM Analysis**

**Kevin K. D. Tan^1,2^, Mark A. Tsuchida^2^, Jenu V. Chacko^2^, Niklas A. Gahm^1,2,3^ and Kevin W. Eliceiri^1,2,3,4*^**

^1^Department of Biomedical Engineering, University of Wisconsin, Madison, WI 53706

^2^Center for Quantitative Cell Imaging, University of Wisconsin, Madison WI 53706-1205

^3^Morgridge Institute for Research, Madison, WI 53715-1119

^4^Department of Medical Physics, University of Wisconsin, Madison, WI 53706

^*^Corresponding author

E-mail: [eliceiri@wisc.edu](mailto:eliceiri@wisc.edu)

# Supplementary Material: DataSheet 1

## Benchmarking

To find an appropriate real-time methodology which can act as a lifetime viewfinder, we compared four common fast-FLIM techniques including Rapid Lifetime Determination (RLD), Phasor Analysis, Laguerre deconvolution, and Noise Corrected Principal Component Analysis (NC-PCA). We benchmark these four different methods in the repository: <https://github.com/uw-loci/RT-FLIM>. NC-PCA is of interest since it corrects for the Poisson distribution of noise in the image and principal component analysis is a computationally fast analysis that can quickly provide an estimated relative lifetime score (i.e., not the actual lifetime, but an estimate of the pixel lifetime variance from mean for the image) (1). Additionally, phasor analysis is of interest since it comes from frequency domain FLIM and acts globally on all pixels in an image. This method estimates the phase and modulation of each fluorescent molecular species from time-domain data and can separate lifetime distributions by projecting them into a two-dimensional space. This two dimensional projection can then be further used to give a lifetime score to each pixel for fast grouping of clusters and with slower computation be used to calculate the actual lifetimes present in individual pixels (2). Laguerre deconvolution based approaches function similarly to a phasor approach, but rather than projecting into the frequency domain, they project into Laguerre space, from which a scoring of the lifetimes of pixels in the image can be generated for fast estimation (3). Furthermore, RLD provides a general description of the lifetime decay process and can come in multi-component variants (4). It provides a significantly faster calculation methodology than a least squares fitting approach and has been shown to provide a fairly accurate calculation of lifetime under most conditions (4). All methods were compared to each other and to the gold standard LMA (1,3–15). The comparisons were performed on simulated and experimental data and the MATLAB code is available at <https://github.com/uw-loci/RT-FLIM>.

## Installation steps for the Napari-Live-FLIM plugin on an OpenScan-LSM system

1. OpenScan TCSPC module and all dependencies must be present.
   1. Verify FLIM electronics are compatible with OpenScan.
      Refer https://github.com/openscan-lsm/OpenScan
2. Python and the following packages must be present.
   1. FLIMLib (from github.com/flimlib). This package is automatically installed when Napari-Live-FLIM is installed.
   2. Napari package and all dependencies (see napari.org)
   3. Napari-Live-FLIM (from github.com/uw-loci/napari-live-flim)
3. In MicroManager, set a port number in the device property setting named **OpenScanFLIM-BH-TCSPC-SendFLIMHistogramsToUDPPort (**Figure S1)
4. In Napari, select **Plugins > FLIM Viewer (Napari-Live-FLIM)** to run the plugin. Enter the same port number to connect to OpenScan.
5. Begin acquisition within MicroManager.
6. Interact with the FLIM data in real-time within napari.
   1. Modify the FLIM Parameters and Display Filters settings as desired.
   2. Add selections to the lifetime Image or phasor plot by clicking the relevant New Selection buttons.
   3. Manipulate the selections with the mouse cursor and modify the selection layer with the layer controls.
   4. Click the Snapshot button during acquisition to take a snapshot.
   5. Use the scroll bar under the lifetime image to recall a specific snapshot.
7. Stop scanning within MicroManager to end acquisition.


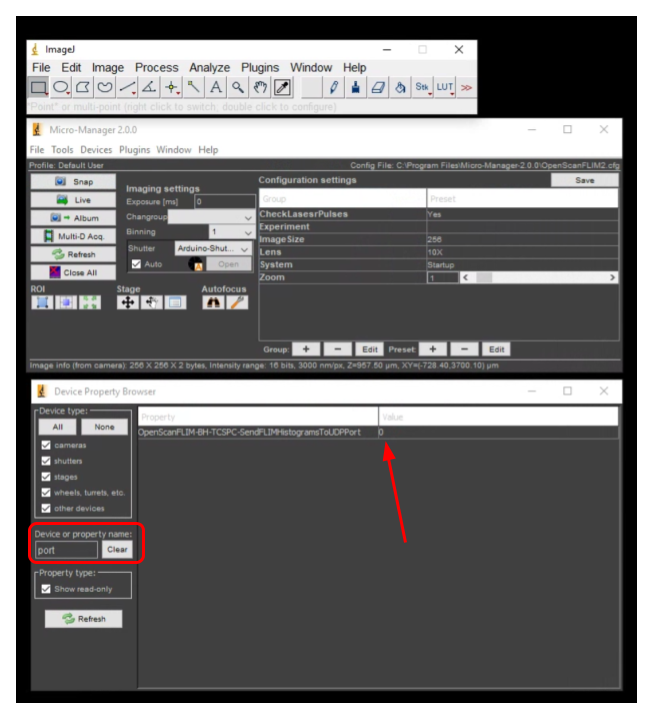


**Figure S1. Screenshot of MicroManager showing how to set up FLIM data output**

# Data Availability

The original contributions presented in the study are publicly available. Instructions for usage with OpenScan are provided above.

The data can be found here:

- GitHub:
  - <https://github.com/uw-loci/napari-live-flim>
  - <https://github.com/uw-loci/RT-FLIM>
  - <https://github.com/flimlib/flimlib>
  - <https://github.com/uw-loci/flimlib_python_examples>
- Zenodo: <https://zenodo.org/records/10019525>
  - Video 1: [Demonstration of Initializing Napari-Live-FLIM](https://zenodo.org/record/7261293/files/Demo_Initializing.mkv?download=1)
  - Video 2: [Demonstration of Real time Acquisition](https://zenodo.org/record/7261293/files/Demo_workingRealtime.mkv?download=1)
  - Video 3: [Demonstration of Replay of SPC file and visualization in Napari-Live-FLIM](https://zenodo.org/record/7261293/files/Demo_ReplaySPC.mkv?download=1)
  - [Example Dataset (for replay test)](https://zenodo.org/record/7261293/files/OpenScan-BHSPC_0077.zip?download=1) in Zenodo
- Additional resources:
  - <https://pypi.org/project/flimlib/>
  - <https://www.napari-hub.org/plugins/napari-live-flim>

# Supplementary Video placeholders:


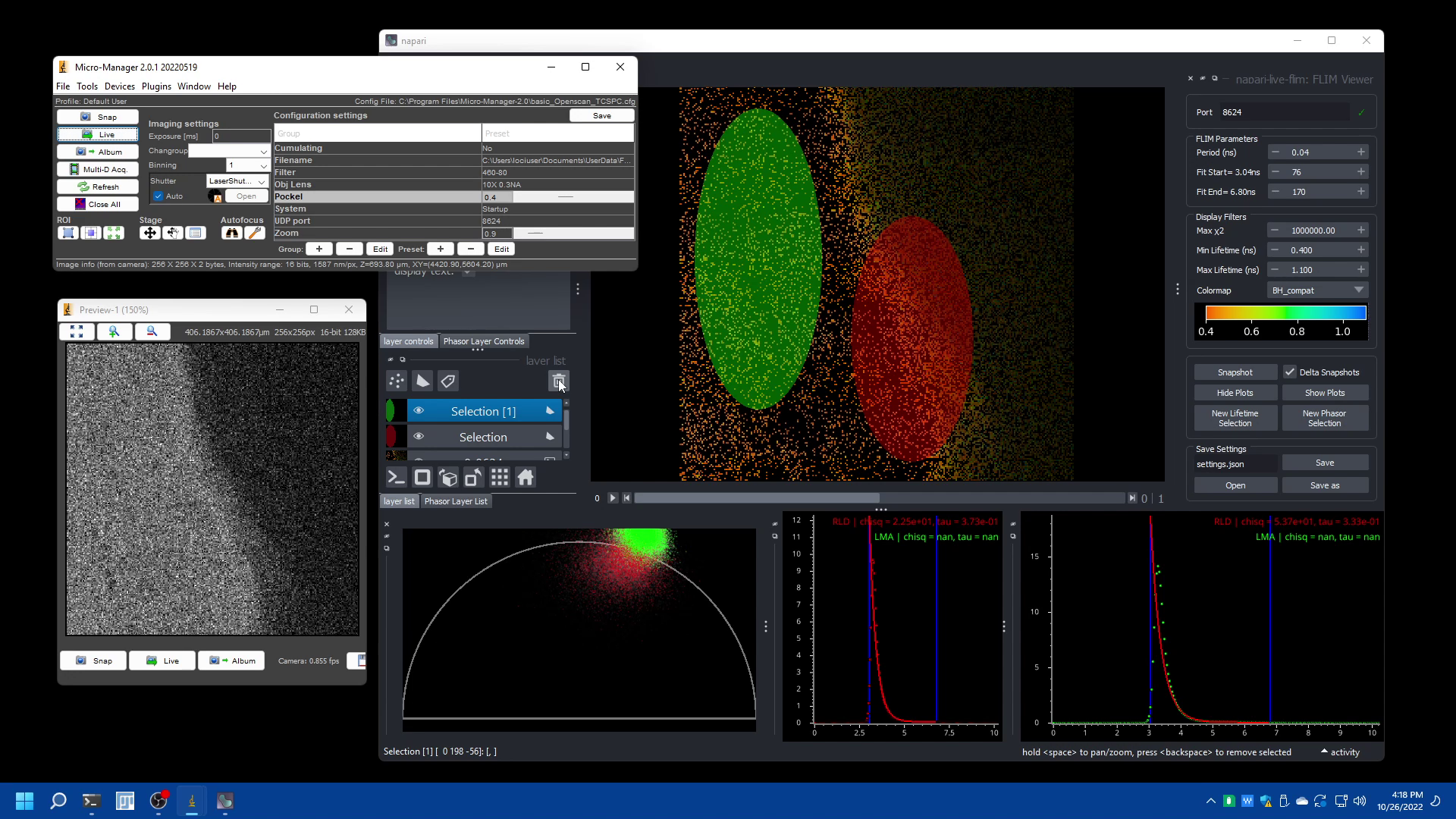


Video 1:

[Demonstration of Real time Acquisition](https://zenodo.org/record/7261293/files/Demo_workingRealtime.mkv?download=1)


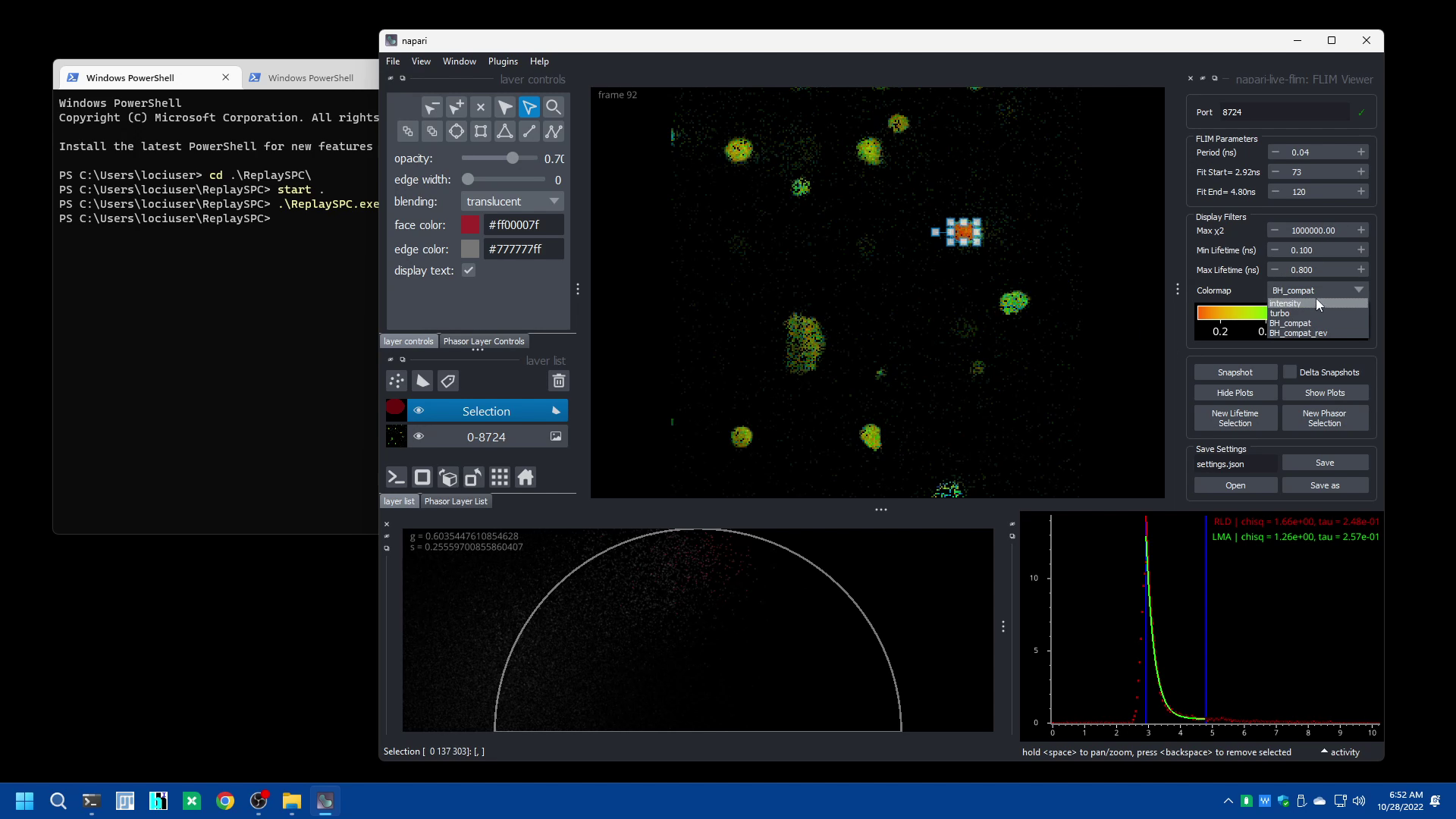


Video 2:

[Demonstration of Replay of SPC file and visualization in Napari-Live-FLIM](https://zenodo.org/record/7261293/files/Demo_ReplaySPC.mkv?download=1)


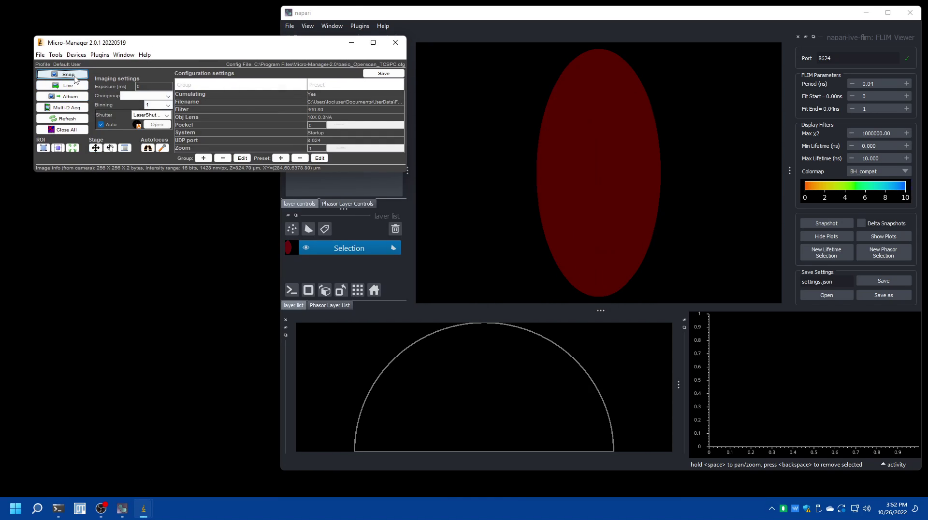


Video 3: Initializing the collection OpenScan and Napari Live FLIM Viewer
[Demonstration of Initializing Napari-Live-FLIM](https://zenodo.org/record/7261293/files/Demo_Initializing.mkv?download=1)

# References

1. Le Marois A, Labouesse S, Suhling K, Heintzmann R. Noise-Corrected Principal Component Analysis of fluorescence lifetime imaging data. Journal of Biophotonics. 2017;10(9):1124–33.

2. Digman MA, Gratton E, Marcu L, French P, Elson D. The phasor approach to fluorescence lifetime imaging: Exploiting phasor linear properties. In: Fluorescence lifetime spectroscopy and imaging. CRC Press; 2014. p. 235–48.

3. Fereidouni F, Gorpas D, Ma D, Fatakdawala H, Marcu L. Rapid fluorescence lifetime estimation with modified phasor approach and Laguerre deconvolution: a comparative study. Methods Appl Fluoresc. 2017 Sep 1;5(3):035003.

4. Sharman KK, Periasamy A, Ashworth H, Demas JN. Error Analysis of the Rapid Lifetime Determination Method for Double-Exponential Decays and New Windowing Schemes. Anal Chem. 1999 Mar 1;71(5):947–52.

5. Levenberg K. A method for the solution of certain non-linear problems in least squares. Quart Appl Math. 1944;2(2):164–8.

6. Ballew RM, Demas JN. An error analysis of the rapid lifetime determination method for the evaluation of single exponential decays. Anal Chem. 1989 Jan 1;61(1):30–3.

7. Jo JA, Fang Q, Marcu L. Ultrafast method for the analysis of fluorescence lifetime imaging microscopy data based on the Laguerre expansion technique. IEEE Journal of Selected Topics in Quantum Electronics. 2005 Jul;11(4):835–45.

8. Digman MA, Caiolfa VR, Zamai M, Gratton E. The Phasor Approach to Fluorescence Lifetime Imaging Analysis. Biophysical Journal. 2008 Jan 15;94(2):L14–6.

9. Pande P, Jo JA. Automated Analysis of Fluorescence Lifetime Imaging Microscopy (FLIM) Data Based on the Laguerre Deconvolution Method. IEEE Transactions on Biomedical Engineering. 2011 Jan;58(1):172–81.

10. Zhu X, Zhang D. Efficient Parallel Levenberg-Marquardt Model Fitting towards Real-Time Automated Parametric Imaging Microscopy. PLOS ONE. 2013 Oct 10;8(10):e76665.

11. Liao SC, Sun Y, Coskun U. FLIM Analysis using the Phasor Plots. ISS Inc: Champaign, IL, USA. 2014;61822:13.

12. Lakner PH, Monaghan MG, Möller Y, Olayioye MA, Schenke-Layland K. Applying phasor approach analysis of multiphoton FLIM measurements to probe the metabolic activity of three-dimensional in vitro cell culture models. Scientific Reports. 2017 Feb 13;7(1):42730.

13. Ranjit S, Malacrida L, Jameson DM, Gratton E. Fit-free analysis of fluorescence lifetime imaging data using the phasor approach. Nat Protoc. 2018 Sep;13(9):1979–2004.

14. Silva SF, Domingues JP, Morgado AM. Can we use rapid lifetime determination for fast, fluorescence lifetime based, metabolic imaging? Precision and accuracy of double-exponential decay measurements with low total counts. Maitland KC, editor. PLoS ONE. 2019 May 14;14(5):e0216894.

15. Gavin HP. The Levenberg-Marquardt algorithm for nonlinear least squares curve-ﬁtting problems. 2020;19.
